# Clinical and Epidemiological Aspects of Late Onset Multiple Sclerosis in East-Azerbaijan, Iran; A Population-Based Study

**DOI:** 10.34172/aim.2022.114

**Published:** 2022-11-01

**Authors:** Ehsan Nasiri, Amirreza Naseri, Pouya Abbasgholizadeh, Ali Fahidi, Mahnaz Talebi

**Affiliations:** ^1^Student Research Committee, Tabriz University of Medical Sciences, Tabriz, Iran; ^2^Neurosciences Research Center (NSRC), Tabriz University of Medical Sciences, Tabriz, Iran

**Keywords:** Elderly, Epidemiology, Late-onset multiple sclerosis, LOMS, Multiple sclerosis

## Abstract

**Background:**

Late-onset multiple sclerosis (LOMS) is defined as symptoms initiating at an age above 50.

**Objective:**

This study aims to determine the clinical and epidemiological aspects of LOMS in East-Azerbaijan province, Iran.

**Methods:**

This population-based study recruited nearly all MS patients to the end of 2020, who were diagnosed at age≥50, by referring to the only local MS registry center. We investigated prevalence, sex, age-of-onset, first clinical presentation, family history, and gap of diagnosis. Also, we compared the disease characteristics between male and female cases.

**Results:**

Out of 4905 total cases of MS, 217 cases (4.42%) were LOMS. The mean age of onset was 53.80±3.41 years with a maximum age of 68 years. The most common age group of the patients was 50 to 55 years (69.1%). The frequency by sex of LOMS in females (150) was greater than males (67). Positive family history was seen in 6.17%, and in 41% of the patients, the disease was diagnosed in a timely manner. Early symptoms were motor (31.3%), sensory (24.8%), optic neuritis (23%), cerebellar symptoms (13.8%), and brainstem symptoms (6.9%). The first presentation of the disease was different between male and female cases (*P*-value<0.01). Motor symptoms were the most prevalent first clinical presentation in female cases (37.6%), while in male cases, cerebellar symptoms (25.8%) were the most common.

**Conclusion:**

LOMS is not a rare condition. Increasing knowledge in the diagnosis, as well as increasing awareness of the disease in the general population, leads to early diagnosis of LOMS and prevention of consequences.

## Introduction

 Multiple sclerosis (MS) is a chronic autoimmune inflammatory disease of the central nervous system which can cause disability.^1^ In most cases, MS involves young adults at 20 to 40 years of age.^2^ In late-onset multiple sclerosis (LOMS), the symptoms initiate at an age of 50 years, which constitutes about 5% of the cases.^3^ There is disagreement among articles regarding the cut-off for LOMS, but most of them agree on 50 years of age.

 Although there are multiple studies regarding the clinical characteristics of adult-onset MS, studies concerning the clinical characteristics of LOMS are limited.^4^ Many similar manifestations between LOMS and other diseases of the elderly lead to a remarkable diagnostic gap and misdiagnosis.^5,6^ Some literature found a poorer prognosis for LOMS which can suggest intensive treatment starting at earlier stages of the disease progression.^5^

 An accurate understanding of the pathogenesis of the disease, as well as accurate diagnosis and treatment of LOMS, is important. For this purpose, more information on the epidemiology, clinical presentation, and prognosis of the disease should be provided. A systematic review of clinical features of LOMS showed that in 49.80% of the cases, the phenotype is relapsing-remitting MS (RRMS) which suggested higher prevalence of progressive MS in LOMS cases. Although MS is still more prevalent in females, this study found a trend of rising proportions of male cases with aging.^3^

 The epidemiologic data on LOMS in the Middle East and Iran is rare. This study aims to determine the prevalence of LOMS in northwestern Iran. Also, demographic and clinical characteristics, family history, and the disease diagnostic gap were evaluated.

## Materials and Methods

 The study was conducted in the East-Azerbaijan (EA) province, which is located in northwestern Iran.

###  Case Ascertainment

 This study was performed by reviewing the patients’ files in the only MS registry center of the province in the university hospital of the TUOMS, affiliated to the Ministry of Health and Medical Education (MOHME). This MS Center was launched in 2008. Registration is mandatory for future therapeutic checkups, public insurance laws, and obtaining drug approval; therefore, this database includes almost all MS patients, even those who were diagnosed before 2008, in the EA province.

 Diagnosis was made according to the 2005 version of McDonald criteria for patients until 2011; patients from 2011 until 2017 were diagnosed using the 2010-revised version of McDonald criteria, and from 2017 forward using the 2017-revised version. All patients were diagnosed by experienced neurologists. MRI (1.5 Tesla) was performed for all patients. All subtypes of MS (relapsing-remitting, secondary progressive, and primary progressive) were covered in this study. The following tests were requested to rule out other diseases, if necessary: anti-phospholipids and anti-cardiolipin immunoglobulin G (IgG) and immunoglobulin M (IgM) antibodies, erythrocyte sedimentation rate (ESR), C-reactive protein (CRP), anti-nuclear antibodies, anti-double-stranded DNA (anti-dsDNA), anti-neutrophil cytoplasmic antibodies, angiotensin-converting enzyme inhibitors, serum B12 level, and other laboratory tests.

 This study includes patients in whom MS started at age ≥ 50 years to the end of 2020. In cases of lack of complete file information, patients were contacted by phone. In this study, we considered the age of the disease onset, sex, first clinical presentations, family history, and diagnostic gap. Gap of diagnosis is the interval between the first presentation of the disease to confirming the diagnosis, so timely diagnosis is defined as the diagnosis of MS in the first days of symptom presentation. First disease presentation is classified as motor, sensory, optic neuritis, cerebellar symptoms, and brainstem symptoms. Cranial nerve involvement (such as double vision, facial weakness, and numbness) and vestibular symptoms (such as vertigo) are classified as brainstem symptoms and ataxia is considered as a cerebellar symptom.

###  Statistical Analyses 

 Analysis was conducted using IBM SPSS Statistics 26.0 (SPSS Inc., Chicago, IL, USA) with a 0.05 level of significance for *P *value and 95% confidence intervals (CIs). Descriptive statistics were reported in percent or mean ± standard deviation, median and interquartile ranges (IQR). Independent sample *t* test, Mann-Whitney U test, and chi-square were used for comparison between male and female cases. Kolmogorov-Smirnov test was utilized for evaluating the normality of the data. All of the reported prevalence values were estimated in 100 000 of the total population with 95% CIs.

## Results

 The total number of MS patients in EA by the end of 2020 was 4905, consisting of 4016 females (81.8%) and 889 males (18.1%). A total of 217 cases (4.4%) were LOMS which included 150 females (3.7% of all female cases) and 67 males (7.5% of all male cases). The frequency of LOMS was higher in females, with a 2.2:1 female-to-male ratio.

###  Prevalence

 The age- and sex-specific prevalence rates of the disease are presented in Table 1. The overall regional prevalence of LOMS was 27.06 (95% CI: 23.69-30.91), including 16.80 (95% CI: 13.22–21.34) in males and 37.22 (95% CI: 31.72–43.68) in females (prevalence is reported per 100 000 population). The number of patients diagnosed between 50 to 60 years of age was 203 (out of 217) which comprised 93.5% of the LOMS cases. Also, 69.1% of the patients were diagnosed between 50 to 55 years of age.

**Table 1:** Age- and Sex-Specific Prevalence of Late-Onset Multiple Sclerosis in East-Azerbaijan, Iran

**Age Group**	**Males**				**Females**				**Both Sexes**			
	**Population**	**No. of** **cases**	**per** **100000**	**95% CI**	**Population**	**No. of** **cases**	**per** **100000**	**95% CI**	**Population**	**No. of** **cases**	**per** **100000**	**95% CI**
50–54	103 574	47	45.38	34.10-60.39	101 132	103	101.85	83.97-123.53	204 706	150	73.28	62.44-85.99
55–59	87 340	16	18.32	11.22-29.90	90 258	37	40.99	29.70-56.58	177 598	53	29.84	22.80-39.06
60–64	67 249	3	4.46	1.44-13.83	69 269	9	12.99	6.76-24.97	136 518	12	8.79	4.99-15.48
65–69	44 740	1	2.24	0.31-15.87	48 694	1	2.05	0.29-14.58	93 434	2	2.03	0.51-8.12
≥ 70	96 025	0	—	—	93 617	0	—	—	189 642	0	—	—
Total	398 928	67	16.80	13.22-21.34	402 970	150	37.22	31.72-43.68	801 898	217	27.06	23.69-30.91

CI: confidence interval. Note: Populations were retrieved from the latest national census and number of cases was based on the reported disease-onset age in the only multiple sclerosis registry of the province.

###  Characteristic of the Patients

**Figure 1:**
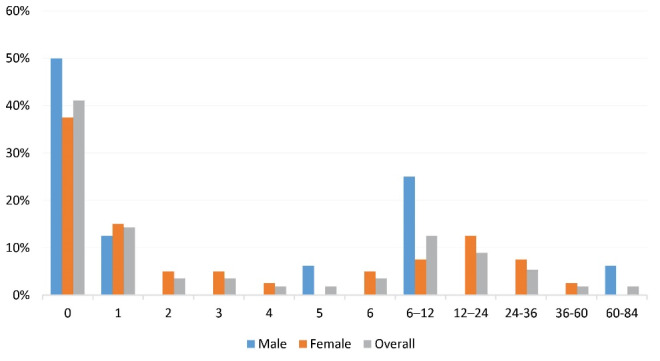
Frequencies of Diagnostic Gaps (in Months).

Table 2 summarizes the characteristic of the patients, as well as comparing female and male cases. The mean age of the disease onset was 53.80 ± 3.41 (median: 52; IQR: 5) years, with a maximum age of 68 years, which was not different between female and male patients (*P* = 0.15). The mean gap of diagnosis was 8.73 ± 15.91 (median: 0; IQR: 0) months, 6.1% of the patients had positive familial history and there was not a significant difference between male and female cases in this regard (*P* = 0.98). Also, there was not a significant difference between male and female cases in terms of the mean gap of diagnosis (*P* = 0.47). As shown in Figure 1, in 41% of the patients, the disease was diagnosed in a timely manner.

**Table 2:** Summary of Characteristics of Late-Onset Multiple Sclerosis Cases in East-Azerbaijan, Iran

**Characteristics **	**All Cases (N=217)**	**Males (n=67)**	**Females (n=150)**	***P*** ** Value**
Age at diagnosis (years)	53.80 ± 3.41	54.15 ± 3.04	53.63 ± 3.58	0.15
Gap of diagnosis (months)	8.73 ± 15.91	8.67 ± 20.73	8.75 ± 13.84	0.47
Familial history	6.17%	6.12%	6.19%	0.98

###  First Clinical Presentation

**Figure 2:**
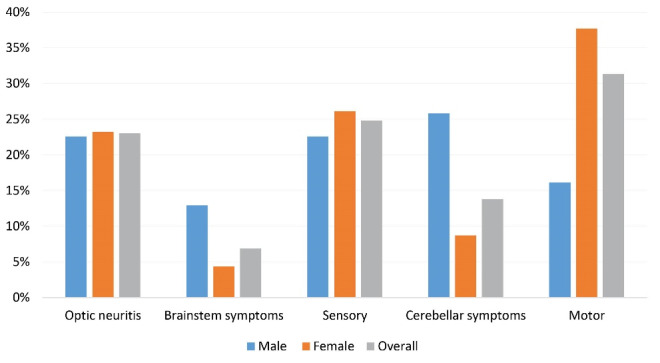
First Symptom of the Disease in Late-Onset Multiple Sclerosis Cases.

 The reported early symptoms were motor (31.3%), sensory (24.8%), optic neuritis (23.0%), cerebellar symptoms (13.8%), and brainstem symptoms (6.9%). The first clinical presentations in male and female cases are shown in Figure 2. There was a significant difference between male and female cases in terms of first disease presentation (*P* < 0.01). The most common first clinical presentation was cerebellar symptoms among male patients (25.8%) and motor symptoms among female cases (37.6%).

## Discussion

 In this study, we described the prevalence of LOMS in EA, Iran which was 27.06 (95% CI: 23.69–30.91) per 100 000 population, including 16.80 (95% CI: 13.22–21.34) per 100 000 males and 37.22 (95% CI: 31.72–43.68) per 100 000 females. Moreover, 217/4905 (4.4%) of MS cases in this area were LOMS. The prevalence of LOMS among females was higher in every onset age group and also in total, such that 69.1% of LOMS cases were females. The peak prevalence of disease diagnosis was in the 50-54 age group. Positive family history was seen in 6.1% of patients. Motor symptoms were the most prevalent first clinical presentation in total and female cases, but in male cases, cerebellar symptoms were the most frequent. Based on our findings, there was a gap of diagnosis in about 60% of LOMS cases, which was up to 7 years. Timely diagnosis was achieved in 40% of the LOMS cases.

 In a study conducted in Canada,^7^ the number of LOMS patients versus all MS patients was 358/5985 (6%) which is slightly higher than our findings. In another study conducted in Spain,^8^ the number of LOMS patients versus all MS patients was 18/368 (4.8%). Moreover, in another study in France,^9^ the proportion of LOMS was 3.4% (46 /1417). In our study, this rate was 4.4% which is similar to these two studies.

 The mean age of patients have been reported at 55.3 ± 4.45,^10^ 74 ± 4.9,^11^ 53.5 ± 3.1,^12^ 55.1 ± 4.3^13^ in Canada, Denmark, Israel, and Iran, respectively. Compared to our study, these findings are similar except for the study conducted in Denmark which can a point of further investigation in future studies in this region.

 In another study of this research project, we found the prevalence of MS by the end of 2017 at 75.72 per 100 000 in the EA province. This study also reported a female-to-male ratio of 3:1, so that 74.32% of the cases were females. The sex-specific prevalence rate was 38.2 in men (95% CI: 35.5–41.0), and 114.61 in women (95% CI: 109.9–119.4) and the rate of positive family history was 13.9% in the mentioned study.^14^ With a special focus on LOMS cases in this study, although there was not a considerable difference in terms of female ratio, we found a smaller proportion of patients with positive family history. The rate of positive family history in other LOMS studies ranges from 8.2% in Australia^15^, and 1.8% in the first degree, and 0.4% in the second degree in Sweden.^16^ Also, a 12.5% rate of family history in Isfahan^17^ and 10.1% in Kuwait^18^ have been reported in geographic locations similar to our study. Our study showed that 6.1% of cases had a positive family history of MS which was lower than the mentioned studies.

 A retrospective study in the Isfahan province of Iran showed that the proportion of females in all cases was 70.2%.^17^ A recent systematic review showed that the overall proportion of female cases to all the cases in studies with the cut-off of 50 years for LOMS was 64.46%.^3^ In this study, we found out that the proportion of females to all cases was 69.1% which is similar to the findings of recent studies, but is lower than the 77.35% rate of females in the Fars province of Iran.^19^ In a study by de Campos Lotti et al, the female-to-male proportion was reported as 19/10 (1.9),^20^ and in a study by Lavandier et al, the proportion of females to males was 17/8 (2.12)^21^; moreover, in a study by Cossburn et al, the proportion of females to all cases was 24/25 (0.96)^22^ which is higher than our recent findings.

 Kis et al noted that the first presentation of LOMS is motor symptoms (80% of the cases), followed by sensory symptoms (45%), ataxia (25%), oculomotor symptoms (5%), and visual disturbance (5%).^23^ In the article by Guillemin et al the first disease presentation was isolated long tracts dysfunction (61.6%), isolated brainstem dysfunction (6.5%), and isolated optic neuritis (5.7%).^24^ However, in another article by D’Amico et al the first disease presentation was spinal symptoms (42.7%), supratentorial symptoms (38.5%), brainstem/cerebellar symptoms (1.4%), and visual symptoms (17.4%).^25^ A systematic review of the clinical features of LOMS reported motor symptoms as the most common initial presentation of LOMS such that its proportion ranges between 35.4% to 100% in different studies. Sensory problems were the second most prevalent symptom (range: 5% to 94%) and visual symptoms (range: 5% to 22.9%), followed by brainstem dysfunction (range: 12.3% to 25%).^3^ Motor and sensory symptoms were the most prevalent first disease presentations in the LOMS cases of our study, which is in line with the recent findings.

 A meta-analysis of common clinical symptoms found fatigue, motor symptoms, and balance dysfunction as the most common symptoms in Iranian MS patients.^26^ Among Iranian LOMS cases, Mirmosayyeb et al in 2020, found motor symptoms (38.1%,) and sensory disturbances (31.0%) to be the most common symptoms in MS patients of Isfahan city.^17^ Taken together with our findings, it seems that there is a difference between the first clinical symptoms of adult-onset and LOMS.

 In conclusion, LOMS is not rare in northwestern Iran. With a considerable gap of diagnosis which is (on the average) 8.73 months, health professionals should take into consideration the symptoms in the elderly. Increasing knowledge in the diagnosis of MS, better access to MRI, revisions of diagnostic criteria, and increasing awareness of the disease in the general population lead to early diagnosis and rising disease prevalence. Lack of reliable data regarding the type, duration, and severity of MS based on the expanded disability status scale, were the limitations of this study, which can be addressed in future investigations.
